# The hows and whys of amastigote flagellum motility in *Trypanosoma cruzi*

**DOI:** 10.1128/mbio.00531-23

**Published:** 2023-06-06

**Authors:** Aline Araujo Alves, Philippe Bastin

**Affiliations:** 1 Trypanosome Cell Biology Unit, Institut Pasteur, Université de Paris Cité, INSERM U1201, Paris, France; Yale University School of Public Health, New Haven, Connecticut, USA

**Keywords:** *Trypanosoma cruzi*, amastigote, flagellum, motility, host-parasite interaction

## Abstract

The protist *Trypanosoma cruzi* exhibits several extracellular stages characterized by the presence of a long and motile flagellum and one intracellular life cycle stage termed amastigote, which possesses a tiny flagellum barely exiting the flagellar pocket. This stage was so far described as replicative but immotile cells. Unexpectedly, the recent work of M. M. Won, T. Krüger, M. Engstler, and B. A. Burleigh (mBio 14:e03556-22, 2023, https://doi.org/10.1128/mbio.03556-22) revealed that this short flagellum actually displays beating activity. This commentary explores how such a short flagellum could be constructed and why it could affect the parasite’s survival inside the mammalian host.

## THE SURPRISE

*Trypanosoma cruzi*, the causative agent of Chagas disease, is a flagellated parasite with a complex life cycle between two hosts. It passes through three major stages: the epimastigote, present in the insect host; the trypomastigote, found in the bloodstream of the mammalian host; and the amastigote, present inside the mammalian host cells. The epimastigote and trypomastigote forms have a long motile flagellum, with the canonical 9+2 axoneme. *T. cruzi* and most trypanosomatid parasites have an extra-axonemal structure, the paraflagellar rod (PFR), which is present in these two life cycle stages and is required for cell motility in epimastigote (1). By contrast, the amastigote form is small and has a short flagellum, with an average length of 2.7 µm and a distal end outside the flagellar pocket. After more than a century of investigations, the recent work of Won and colleagues brings to light the unexpected motile activity of the amastigote short flagellum. They describe the flagellar beats in a quasiperiodic behavior with a variable beating frequency and a rotational movement, showing motility as an intrinsic property of the amastigote flagellum. This result opens several questions, such as (i) how is this short flagellum formed during differentiation from trypomastigote? and (ii) why does amastigote keep a motile flagellum?

**Fig 1:**
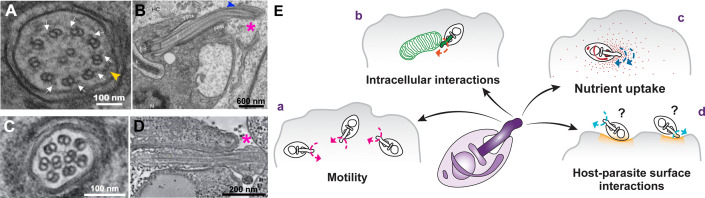
The *Trypanosoma cruzi* amastigote short flagellum: structure and possible roles in parasite survival. *T. cruzi* amastigote form (**A, B**) has a canonical axoneme, with a central pair and dynein arms (white arrows) (**A**). A candidate IFT particle can be observed (yellow arrowhead). *T. cruzi* amastigote flagellum has a distal portion outside the flagellar pocket (**B**, pink asterisk) (6). An electron-dense structure resembling the PFR is indicated with a blue arrowhead. In comparison, *L. mexicana* shows a 9+0 axoneme with no dynein arms (**C**), that has only a minor portion exiting the flagellar pocket (**D**, pink asterisk) (7). In (**E)** we propose different functions the motile short flagellum could have in amastigote interaction with the mammalian host.

How can the amastigote short flagellum have an active beating? *T. cruzi* amastigote maintains elements of a motile 9+2 axoneme such as dynein arms and central pair apparatus, as shown by structural (Fig. 1A and B) and biochemical approaches (2 - 4), arguing in favor of a motile activity. In amastigote, it is commonly reported that the PFR is absent. Intriguingly, when observed by electron microscopy, the amastigote flagellum exhibits some electron-dense material at the same position where the PFR is observed in other stages (Fig. 1A), suggesting the existence of a reduced PFR. Moreover, proteome data reveal the presence of PFR proteins, such as PFR2 and PAR4, in amastigote forms (2, 4). In epimastigote forms, where the PFR is present, the flagellar beat shows a different pattern with asymmetric base-to-tip waves (5). Won and colleagues argue that the quasiperiodic beating pattern observed in the amastigote flagellum can be given by its short length, but how much impact does a reduced or possibly absent PFR have on these differences in wave forming?

## How is the short motile flagellum constructed?

How could such a short flagellum be put in place during differentiation? Does it originate from the shortening of the trypomastigote flagellum or *de novo* assembly, as debated for *Leishmania* (8)? Assembling a flagellum relies on the well-conserved transporting mechanism called intraflagellar transport (IFT), which delivers the building elements to the tip of the growing flagellum (9). A dense structure between the axoneme and the flagellar membrane that resembles an IFT particle is actually observed on the amastigote flagellar section (Fig. 1A, arrowhead). This morphological data is strengthened by the presence of some IFT proteins (IFT81, IFT88, and IFT20) identified in amastigote during the flagellum membrane protein SMP1-1 proximity proteome (4).

If the amastigote flagellum is assembled by IFT, why is it so short? Several models can be proposed. The first one is based on what was described in the *T. brucei* procyclic form that uses a grow-and-lock model to prevent further elongation of the existing flagellum (10). One could imagine a premature locking leading to the formation of a very short flagellum. The second is the “balance-point” model, where the axoneme length is determined by the equilibrium between flagellum assembly and disassembly (11). That would suggest that the amastigote flagellum is less stable or at least has a faster turnover when compared to other stages. A third model would be the limitation of the pool of soluble tubulin or associated material.

## Other short flagella, different functions?

The presence of a life cycle stage with a short flagellum is not exclusive to *T. cruzi,* and it is observed in at least two other cases. The first one is the amastigote stage of *L. mexicana*. They are morphologically close to *T. cruzi* amastigotes and are also found inside the host cell; however, their short flagellum (Fig. 1C and D) lacks the dynein arms and the central pair (7) and presumably is immotile. Therefore, although *L. mexicana* and *T. cruzi* share a morphologically similar amastigote life cycle stage, their functions are clearly different. This opens the discussion of how these differences can be related to specific interactions between these parasites and their host cells.

The second case is the *T. brucei* short epimastigote found in the cardia of the insect host, the tsetse fly. The short epimastigote keeps a short motile flagellum that has the canonical axoneme and the PFR. Moreover, it has been proposed that this form is responsible for the invasion of the salivary glands and that the short flagellum could be responsible for anchoring to the epithelium, possibly via a sensory mechanism (12, 13). This form also keeps an active IFT system that could be responsible for the flagellum elongation during differentiation to the attached epimastigote stage (14). This situation is therefore more akin to that of the *T. cruzi* amastigote.

## Why would the amastigote stage need motility?

Is the amastigote parasite able to move in the cytosol of host cells? It was observed in the alga *Chlamydomonas reinhardtii* that a flagellum longer than 2 µm can generate a rotation force in the cell body (15). Given that, the amastigote flagellum could, in theory, power cell movement (Fig. 1E–a), although that has not been reported yet. In this context, the *T. cruzi* flagellum could be guided by sensing means, as this organelle is considered a sensory platform since it contains different signaling proteins localised to its membrane like the putative calcium-sensor FCaBP (16) and the cation channel TcCat (17). The close contact of amastigote flagellum with host cell mitochondria was already reported, and a sensory role was speculated at the time (18). Motility could contribute to this interaction, with cell movement possibly helping the search for host organelle targets through signaling molecules (Fig. 1E–b). Flagellar motility could also guide amastigote parasites when searching for and uptaking nutrients within the host cell (Fig. 1E–c). In that matter, it is noteworthy that *T. cruzi* intracellular amastigote has an active endocytic system, in contrast to the extracellular amastigote stage or during differentiation to trypomastigotes (19).

Flagellar movement could also confer advantages for the infectivity of extracellular amastigote. These forms represent 10% of circulating parasites in the blood during acute infection (20), and their capability to infect host cells is well known (21, 22). Could flagellum beating play an active role in generating host cell response to the parasite? Trypomastigote forms can invade host cells by recognition and attachment throughout their flagellum, producing membrane damage that triggers the repairing mechanism by lysosome fusion (23). Even more, the cell body distortions conferred by flagellar movement can also affect infectivity. Trypomastigote shows an active gliding movement while attached to the host cell surface through its posterior region, leading to membrane deformations that help parasite invasion (24). Although extracellular amastigote invades the host cell through a phagocytosis-like pathway, the flagellar beat could confer motility to the cell, helping with the search and recognition of the entry site by a sensory mechanism or even triggering the host cell response by generating movements of the whole cell (Fig. 1E–d).

The amazing discovery of motility in this short flagellum reminds us that, after more than a century of observing *T. cruzi*, we can still have surprises. The finding changes the way we look at the working mechanisms of a well-known structure as the flagellum and turns the amastigote short and active flagellum into an intriguing model to explore flagellum construction and motility. This discovery opens the field to other possible roles the short beating flagellum could play on parasite survival inside and outside the host cell.

## References

[1] Lander N , Li Z-H , Niyogi S , Docampo R . 2015. CRISPR/cas9-induced disruption of paraflagellar rod protein 1 and 2 genes in Trypanosoma cruzi reveals their role in flagellar attachment. mBio 6:e01012. doi:10.1128/mBio.01012-15 26199333 PMC4513075

[2] Atwood JA , Weatherly DB , Minning TA , Bundy B , Cavola C , Opperdoes FR , Orlando R , Tarleton RL . 2005. The Trypanosoma cruzi proteome. Science 309:473–476. doi:10.1126/science.1110289 16020736

[3] Gardener PJ . 1974. Pellicle-associated structures in the amastigote stage of Trypanosoma cruzi and Leishmania species. Ann Trop Med Parasitol 68:167–176. doi:10.1080/00034983.1974.11686935 4212227

[4] Won MM , Baublis A , Burleigh BA . 2023. Proximity-dependent biotinylation and identification of flagellar proteins in Trypanosoma cruzi. mSphere. doi:10.1128/msphere.00088-23 PMC10286712 37017578

[5] Ballesteros-Rodea G , Santillán M , Martínez-Calvillo S , Manning-Cela R . 2012. Flagellar motility of Trypanosoma cruzi epimastigotes. J Biomed Biotechnol 2012:520380. doi:10.1155/2012/520380 22287834 PMC3263639

[6] Won MM , Krüger T , Engstler M , Burleigh BA . 2023. The intracellular amastigote of Trypanosoma cruzi maintains an actively beating flagellum. mBio 14:e0355622. doi:10.1128/mbio.03556-22 36840555 PMC10128032

[7] Gluenz E , Höög JL , Smith AE , Dawe HR , Shaw MK , Gull K . 2010. Beyond 9+0: noncanonical axoneme structures characterize sensory cilia from protists to humans. FASEB J 24:3117–3121. doi:10.1096/fj.09-151381 20371625 PMC2923350

[8] Wheeler RJ , Gluenz E , Gull K . 2015. Basal body multipotency and axonemal remodelling are two pathways to a 9+0 flagellum. Nat Commun 6:8964. doi:10.1038/ncomms9964 26667778 PMC4682162

[9] Prevo B , Scholey JM , Peterman EJG . 2017. Intraflagellar transport: mechanisms of motor action, cooperation, and cargo delivery. FEBS J 284:2905–2931. doi:10.1111/febs.14068 28342295 PMC5603355

[10] Bertiaux E , Morga B , Blisnick T , Rotureau B , Bastin P . 2018. A grow-and-lock model for the control of flagellum length in trypanosomes. Curr Biol 28:3802–3814. doi:10.1016/j.cub.2018.10.031 30449671

[11] Marshall WF , Rosenbaum JL . 2001. Intraflagellar transport balances continuous turnover of outer doublet microtubules: implications for flagellar length control. J Cell Biol 155:405–414. doi:10.1083/jcb.200106141 11684707 PMC2150833

[12] Rotureau B , Subota I , Bastin P . 2011. Molecular bases of cytoskeleton plasticity during the Trypanosoma brucei parasite cycle. Cell Microbiol 13:705–716. doi:10.1111/j.1462-5822.2010.01566.x 21159115

[13] Sharma R , Peacock L , Gluenz E , Gull K , Gibson W , Carrington M . 2008. Asymmetric cell division as a route to reduction in cell length and change in cell morphology in trypanosomes. Protist 159:137–151. doi:10.1016/j.protis.2007.07.004 17931969

[14] Bertiaux E , Mallet A , Rotureau B , Bastin P . 2020. Intraflagellar transport during assembly of flagella of different length in Trypanosoma brucei isolated from tsetse flies. J Cell Sci 133:jcs248989. doi:10.1242/jcs.248989 32843573

[15] Bottier M , Thomas KA , Dutcher SK , Bayly PV . 2019. How does cilium length affect beating? Biophys J 116:1292–1304. doi:10.1016/j.bpj.2019.02.012 30878201 PMC6451027

[16] Buchanan KT , Ames JB , Asfaw SH , Wingard JN , Olson CL , Campana PT , Araújo APU , Engman DM . 2005. A flagellum-specific calcium sensor. J Biol Chem 280:40104–40111. doi:10.1074/jbc.M505777200 16148003

[17] Jimenez V , Docampo R . 2012. Molecular and electrophysiological characterization of a novel cation channel of Trypanosoma cruzi. PLoS Pathog 8:e1002750. doi:10.1371/journal.ppat.1002750 22685407 PMC3369953

[18] Lentini G , Dos Santos Pacheco N , Burleigh BA . 2018. Targeting host mitochondria: a role for the Trypanosoma cruzi amastigote flagellum. Cell Microbiol 20. doi:10.1111/cmi.12807 PMC5764780 29119655

[19] Alcantara CL , de Souza W , Cunha E Silva NL . 2021. The cytostome-cytopharynx complex of intracellular and extracellular amastigotes of Trypanosoma cruzi exhibit structural and functional differences. Cell Microbiol 23:e13346. doi:10.1111/cmi.13346 33900003

[20] Andrews NW , Hong KS , Robbins ES , Nussenzweig V . 1987. Stage-specific surface antigens expressed during the morphogenesis of vertebrate forms of Trypanosoma cruzi. Exp Parasitol 64:474–484. doi:10.1016/0014-4894(87)90062-2 3315736

[21] Bonfim-Melo A , Ferreira ÉR , Mortara RA . 2018. Rac1/WAVE2 and Cdc42/N-WASP participation in actin-dependent host cell invasion by extracellular amastigotes of Trypanosoma cruzi. Front Microbiol 9:360. doi:10.3389/fmicb.2018.00360 29541069 PMC5835522

[22] Walker DM , Oghumu S , Gupta G , McGwire BS , Drew ME , Satoskar AR . 2014. Mechanisms of cellular invasion by intracellular parasites. Cell Mol Life Sci 71:1245–1263. doi:10.1007/s00018-013-1491-1 24221133 PMC4107162

[23] Ferri G , Edreira MM . 2021. All roads lead to cytosol: Trypanosoma cruzi multi-strategic approach to invasion. Front Cell Infect Microbiol 11:634793. doi:10.3389/fcimb.2021.634793 33747982 PMC7973469

[24] Fernandes MC , Cortez M , Flannery AR , Tam C , Mortara RA , Andrews NW . 2011. Trypanosoma cruzi subverts the sphingomyelinase-mediated plasma membrane repair pathway for cell invasion. J Exp Med 208:909–921. doi:10.1084/jem.20102518 21536739 PMC3092353

